# Successful Community Nutrition Incentive Program Data Collection During the COVID-19 Pandemic: A Case Study

**DOI:** 10.1093/cdn/nzac025

**Published:** 2022-02-17

**Authors:** Sarah A Stotz, Hollyanne Fricke, Cameron Perra, Carmen Byker-Shanks, Amy L Yaroch

**Affiliations:** Centers for American Indian and Alaska Native Health, Colorado School of Public Health, University of Colorado Anschutz Medical Campus, Aurora, CO; Gretchen Swanson Center for Nutrition, Omaha, NE; Hunger Task Force, Milwaukee, WI; Gretchen Swanson Center for Nutrition, Omaha, NE; Gretchen Swanson Center for Nutrition, Omaha, NE

**Keywords:** nutrition incentive, program evaluation, fruit and vegetable consumption, qualitative, COVID-19

## Abstract

**Background:**

The COVID-19 pandemic has complicated rigorous evaluation of public health nutrition programs. The United States Department of Agriculture Gus Schumacher Nutrition Incentive Program (GusNIP) funds nutrition incentive programs to improve fruit and vegetable purchasing and intake by incentivizing Supplemental Nutrition Assistance Program (SNAP) participants at the point of sale. GusNIP grantees are required to collect survey data (e.g., fruit and vegetable intake and food insecurity status) on a subset of participants. However, due to COVID-19, most GusNIP grantees faced formidable barriers to data collection. The Hunger Task Force Mobile Market (HTFMM), a Wisconsin-based 2019 GusNIP grantee, employed particularly innovative methods to successfully collect these data (n > 500 surveys).

**Objective:**

To explore HTFMM's successful participant-level data collection evaluation during COVID-19.

**Methods:**

A single case study methodological approach framed this study. The case is HTFMM in Milwaukee, WI, USA. Participants included HTFMM: leadership (n = 3), evaluators (n = 2), staff (n = 3), volunteers (n = 3), and customers (n = 10). These teleconference interviews were recorded and transcribed verbatim. Transcripts were coded using thematic qualitative analysis methods with two independent coders.

**Results:**

Four salient themes emerged. 1) There were multiple key players with unique roles and responsibilities who contributed to personalized, proactive, and time-intensive telephone-based proctored survey collection methods; 2) the importance of resources dedicated to comprehensive evaluation; 3) longstanding relationships rooted in trust and community-based service are key to successful program delivery, engagement, and evaluation; 4) the COVID-19 data collection protocol also serves to mitigate non-pandemic challenges to in-person survey collection.

**Conclusions:**

These findings provide guidance on how alternative methods for data collection during COVID-19 can be employed and applied to other situations which may affect the ability to collect participant-level data. These findings contribute to a growing body of literature as to best practices and approaches to collecting participant-level data to evaluate public health nutrition programs.

## Teaser

This paper details a case study of a mobile fruit and vegetable market and features key facilitators that led to successful public health nutrition program evaluation during the COVID-19 pandemic.

ORIGINAL UNEDITED MANUSCRIPT

## INTRODUCTION

The COVID-19 pandemic has complicated rigorous evaluation of public health nutrition and community nutrition programs throughout the world. Traditional nutrition-specific tools to evaluate public health nutrition and community nutrition programs include surveys on dietary behaviors, 24-hour dietary recall, food records, biometric measures (e.g., body weight), and clinical outcomes (e.g., blood pressure) (1–3). These measures are largely collected in-person, and even without the social distancing precautions related to COVID-19, public health nutrition and community nutrition professionals face challenges with collecting rigorous intervention evaluation data (4,5). Though online data collection for dietary behavior measures is plausible, in some audiences (e.g., older adults, those with limited digital literacy) formidable barriers exist to online dietary behavior data collection (6). Innovative, socially-distanced, and rigorous evaluation methods are required to assess the validity of nutrition programs and interventions. There is little published about successful approaches taken to evaluate these programs during the COVID-19 pandemic. The purpose of this case study is to highlight one public health nutrition program's successful participant-level program evaluation during the COVID-19 pandemic. These findings provide foundation for best practices for future large scale public health nutrition and community nutrition program evaluation during the COVID-19 pandemic and beyond.

### USDA Gus Schumacher Nutrition Incentive Program

The Gus Schumacher Nutrition Incentive Program (GusNIP) provides funding opportunities for organizations across the United States (U.S.) to improve access to fruits and vegetables (FVs) and stimulate local economies (7). GusNIP began in 2019 and is a four-year effort funded by the

ORIGINAL UNEDITED MANUSCRIPT

U.S. Department of Agriculture (USDA) National Institute of Food and Agriculture (NIFA) through the 2018 Farm Bill; GusNIP was predated by the Food Insecurity Nutrition Incentive Program (FINI) from 2014-2018 (7).

GusNIP provides federal funding to implement and evaluate projects that provide incentives to increase the purchase and consumption of FVs by consumers with low income. There are two types of programs under the GusNIP funding mechanism: Produce Prescription (PPR) and Nutrition Incentive (NI) (7). This paper focuses on NI programs. Broadly, GusNIP NI goals are focused on 1) increasing the purchase and consumption of FVs and 2) reducing individual and household food insecurity. GusNIP NI programs seek to increase the purchase of FVs by consumers participating in the Supplemental Nutrition Assistance Program (SNAP), the largest federal food assistance program, by providing incentives for FVs at the point of sale.

Key aspects of NI programs are severalfold. First, qualifying FVs can be any variety of fresh, canned, dried, or frozen whole or cut FVs without added sugars, fats or oils, and salt. Second, incentives are redeemed at the point of sale, including farm direct settings (e.g., farmers markets, mobile markets, community supported agriculture), and brick and mortar (e.g., supermarkets, grocery, corner stores). Finally, nutrition education and/or auxiliary services (e.g., transportation services) are commonly added to assist program participants to more effectively engage in these programs (7). GusNIP NI programs can be administered using a myriad of program designs. For example, a participant can spend $1 with SNAP and subsequently earn $1 for qualifying FVs at participating grocery stores via an electronic discount using a store loyalty card. Other examples

ORIGINAL UNEDITED MANUSCRIPT

of NI program mechanisms include tokens that can be redeemed at farmers markets and farm stands, or discounts offered on community supported agriculture shares, among other models.

Beginning in 2019, the USDA NIFA also funded a National Training, Technical Assistance, Evaluation, and Information Center (NTAE) as part of the GusNIP mechanism, to support grantees in program implementation, reporting, and evaluation. The NTAE developed a set of core metrics that all GusNIP grantees are required to collect. While heterogeneity among programs exists across geography, program design, evaluation, and methodology, shared measures can help elucidate the *national* impact of a large-scale program like GusNIP by aggregating data on key outcomes, including food security and FV consumption (8).

**Hunger Task Force Mobile Market: A 2019 GusNIP NI Grantee**

This case study focuses on the efforts of one NI program to implement programming and conduct evaluation during COVID-19. Because of COVID-19, most GusNIP grantees faced formidable barriers to this participant-level data collection requirement beginning in 2019. However, the Hunger Task Force Mobile Market (HTFMM) employed innovative methods to successfully collect these data.

The HTFMM, based in Milwaukee, WI, USA is a 2019 GusNIP NI program grantee that has leveraged its funding to expand its mobile market reach and offer a 25% discount on all eligible foods. Hunger Task Force is a multi-component food aide program with a long-standing history of community-engaged service to improve food access in the areas it serves (9). Hunger Task Force developed the Mobile Market concept to provide access to FVs to individuals who

ORIGINAL UNEDITED MANUSCRIPT

experience low income and present an alternative to emergency food pantries for people with limited access to food in the city of Milwaukee (9).

As required by all GusNIP grantees, HTFMM collected the required core metrics participant-level survey data throughout the COVID-19 pandemic. HTFMM successfully collected more than 500 surveys, hugely surpassing the NTAE requirement of 150 surveys. The purpose of this case study is to use qualitative inquiry to understand the process, facilitators, and best practices of HTFMM regarding its participant-level survey data collection successes during COVID-19.

## METHODS

### Study Design

This study is framed by a constructivist (10), instrumental, single case-study design, and the unit of analysis is defined as the HTFMM (11). Case study methodology is useful for in-depth formative evaluation of public health programs as it allows researchers to view problems from multiple perspectives and aids to enrich the meaning of a singular perspective (11). In an instrumental case study, the methodology is often used to accomplish something other than understanding a particular situation (e.g., case), in that the case is actually of secondary interest and serves as a supportive role in facilitating understanding of something else (12). In this particular research project, the HTFMM case is used to understand facilitators for successful GusNIP-required participant-level data collection. Due to its flexibility and rigor, the case study approach is valuable for public health research to evaluate programs and develop interventions (13). Ethical approval of this research was obtained from the University of Nebraska Medical

ORIGINAL UNEDITED MANUSCRIPT

Center on March 4, 2021 (IRB # 829-20-EX) and the Institutional Review Board determined it exempt, so no informed consent was required. The study was completed in compliance with waiver of consent, and participants were provided with an information sheet prior to data collection informing them of the purpose of the study.

**Case Study Site**

The HTFMM is a single-aisle grocery store in a car trailer pulled by a truck. Stocked with fresh produce, meat, and dairy, it has coolers with sliding glass doors and stainless-steel shelves for produce displayed in baskets. The HTFMM is scheduled for two 90-minute stops per day at low-income and senior housing developments, community centers, college campuses, and workplaces. While the HTFMM is open to all, older adults make up most customers. Each HTFMM offers up to 50 varieties of locally-sourced produce—as well as meat, dairy, eggs, butter, and juice—at 25% off the grocery partner's lowest store prices. The HTFMM accepts credit and debit cards, including SNAP (known as FoodShare in the state of Wisconsin) (9,14). Images of the HTFMM can be found in **Figure 1**.

**Participants and Recruitment**

Purposive sampling recruitment strategies guided participant recruitment and selection (15). HTFMM leadership, external/internal evaluators, staff, and volunteers were recruited though e-mail invitation and all agreed to participate. HTFMM customers were recruited by verbal invitation from the HTFMM volunteers who had called them to complete a telephonic, proctored survey (GusNIP survey data not part of this dataset). Researchers have no record of the number of customers who declined to participate; however, 3 of the 13 customers who initially agreed

ORIGINAL UNEDITED MANUSCRIPT

were not reachable to schedule the subsequent 1:1 interview. If customers accepted the opportunity to be interviewed for this HTFMM case study, they were scheduled for a subsequent telephone-based interview and received a $15.00 gift card for their time. HTFMM leadership, external/internal evaluators, and staff were not compensated for their time, and participation in the study was not a condition of their employment.

**Data Collection**

One trained qualitative researcher co-author (SAS) interviewed all participants between March-May 2021. Recruitment and data collection concluded when data saturation was reached (16). Zoom was used for HTFMM leadership, evaluators, staff, and volunteer interviews, whereas a telephone-based call-recording application was used for customer interviews (17). The purpose of the latter strategy was to ease the potential technological burden on customers as the researcher was able to call these participants directly on their preferred telephone line as opposed to requesting they log into Zoom. Interviews averaged 36 minutes and ranged from 13-65 minutes in length. The qualitative researcher used a semi-structured interview guide with probes (18), developed in collaboration with HTFMM evaluators and 2 additional qualitative researchers. Moderator guides can be found in **Table 1**.

**Data Analysis**

All recorded interviews were transcribed verbatim by a professional transcription company. After professional transcription, transcripts were checked for accuracy in their entirety by the lead qualitative researcher and deidentified using [NAME]. The research team utilized Atlas.ti (Mac Version 8.1.1) to digitalize and increase transparency in the analytic process (19). Two

ORIGINAL UNEDITED MANUSCRIPT

researchers independently double coded 25% of the transcripts (20). The two coders reached >90% concordance in their independent coding (20). The first round of coding included inductive free coding of two transcripts, where no pre-determined codebook was utilized (20). Coders met after each of these two transcripts were independently coded to discuss the codebook and agree on code definitions. At this point, deductive (*a priori*) codes were also developed based on the moderator guide and were added to the second round of coding. During the second round of coding, the lead qualitative researcher reviewed all transcripts with the agreed upon codebook, including inductive and deductive codes. After all transcripts were coded, researchers summarized and collapsed codes into categories. For example, codes "challenges_literacy," "inclement weather," and "difficult survey questions" were grouped together under category "benefits_proctored surveys" (20). Ultimately, the categories revealed key overarching themes in this thematic analysis (21). The analysis and findings follow the COREQ (Consolidated Criteria for Reporting Qualitative Research) guidelines, a 32-item checklist meant to guide rigorous and systematic reporting of qualitative research (22).

## RESULTS

Findings reflect cross cutting themes from all interviews. Researchers interviewed 100% of the HTFMM leadership (n=3), 100% of the external/internal evaluators (n=2), 75% of the relevant staff (n=3), 80% of volunteers (n=3), and customers (n=10). Four salient themes emerged across these interviews. First, there were multiple key players with unique roles and responsibilities who contributed to personalized, proactive, and time-intensive telephone-based proctored survey collection methods. Second, adequate resources (e.g., expertise and funding) are needed for rigorous evaluation. Third, HTFMM patrons had longstanding relationships rooted in trust with

ORIGINAL UNEDITED MANUSCRIPT

HTFMM, a key factor that motivated them to agree to complete evaluation surveys. Fourth, COVID-19 social distancing data collection protocol also serves to mitigate non-pandemic challenges to in-person survey collection.

**There are multiple key players with unique roles and responsibilities who contributed to personalized, proactive, and time-intensive telephone-based, proctored survey collection methods.** One of the crucial collaboration features between key players included a "warm handoff" from in-person HTFMM staff to telephone-based volunteers who proctor surveys, to avoid "cold calling" customers for survey data collection. Participants shared the unique roles they each played in conducting participant-level program evaluation. One HTFMM leader said:

> *I can't tell you what the key ingredient is – I mean, it's people. It's all of our people – our staff, our University partners, having dedicated customers, it's all what makes these programs and projects work in our community. Having [NAME] to lead the GusNIP evaluation is crucial – and then we do use a lot of volunteers – we couldn't do what we normally do without volunteers every step of the way.*

In lieu of providing additional quotes from respondents about how their individual roles contributed to the success of the data collection procedures, authors have provided an overview of the key roles and responsibilities (see Table 2).

Key roles and responsibilities are summarized in **Table 2.**

ORIGINAL UNEDITED MANUSCRIPT

**Importance of resources dedicated to comprehensive evaluation.** Central to the resources needed to conduct this successful program evaluation was the strategic planning, vision, and support from HTFMM leadership to hire a full-time evaluation expert who had extensive experience in public health program evaluation and public policy analysis. This HTFMM evaluation expert shared:

> *Since last year when we started working on the evaluation projects (…) we really started seriously meeting with [NAME] and others from GusNIP. My role has just been kind of heading up the evaluation side of the Mobile Market in its entirety. That has meant kind of identifying and getting linked up with the researcher side of things so the three researchers that we're working with that another employee at the Hunger Task Force had a connection with and has worked with before on research. (…) We kind of sat down to determine big picture what our research goals we want to get out of this project. How to approach it, the big picture methodology things which was process over some time last summer and then my role has been the implementation. (…) kind of project management of the evaluation has been my role.*

Another key resource included securing a small grant to support evaluation startup costs that were not included in the larger USDA GusNIP grant. This grant (~$6,000) was awarded to collaborating external evaluator academic partners and HTFMM evaluation experts and supported access to required CITI training for all players who handled human subjects research (e.g., recruitment, data collection, data management), data analysis software, and program

ORIGINAL UNEDITED MANUSCRIPT

marketing. Volunteers were also provided with HTFMM cell phones, including paid data plans to support their data collection phone calls. Finally, human resources, which included both paid (e.g., staff) and volunteer time, were crucial to successfully collecting survey data. Details of the time needed by each key player are outlined in **Table 2**.

**Longstanding relationships rooted in trust and community-based service are key to successful program delivery, engagement, and evaluation.** Participants shared many examples of how longstanding relationships between key players have facilitated HTFMM evaluation efforts, especially during the COVID-19 pandemic. For example, customer participants shared sentiments of gratitude for Hunger Task Force as an organization, as well as shared about their robust use of Hunger Task Force resources beyond the mobile market. Customers shared it was this "reputation in our community" that prompted them to participate in the survey. One customer shared her rationale for participating in the survey:

> *I like to help people, especially if they're trying to get information to improve something they've got going. I mean, they're helping me – lots of people like me, and I hope that it's something they can continue. So they need to know if it's [the program]* working or not.

HTFMM leadership and HTFMM external evaluation experts shared that it was their longstanding relationship with university-based academic partners that helped them secure IRB approval (a requirement of all GusNIP grantees and not always easy for community-based organizations to navigate) and the aforementioned supplemental grant to support evaluation start-up costs. HTFMM leadership indicated that they did not experience any delays with establishing

ORIGINAL UNEDITED MANUSCRIPT

this community-academic partnership for their GusNIP project because they had already collaborated on similar evaluation efforts in the past. The HTFMM external evaluator shared their perspective on the challenges community-based organizations may have without longstanding relationships with academic evaluation experts:

> *I think about someone else, if they were trying to establish this academic partnership for the first time (…) I think about those other groups where they suddenly realized they had to do IRB. (…) Because even if they're going to go through IRB on their own, understanding an informed consent document (…). That's a very specialized piece of knowledge that I don't know if all of your grant recipients have access to. Helping them overcome that hurdle of IRB and CITI training and all of those things, I imagine would save time.*

HTFMM evaluators and leadership indicated that longstanding trust, visibility, and community service are key to encourage patrons and participants to participate in evaluation efforts. One HTFMM evaluator explained:

> *They see an organization that they know and trust, they're more likely to trust information or respond to the survey.*

Finally, both HTFMM leadership and volunteers shared the reason their volunteer base was so strong and why volunteers "just keep sticking around" is because of the Hunger Task Force's reputation for quality service to the Milwaukee communities, the variety of opportunities for

ORIGINAL UNEDITED MANUSCRIPT

volunteers (especially prior to COVID-19), and the sense of “contributing to community” that volunteers felt being part of an organization like Hunger Task Force. A HTFMM leader shared:

> *We couldn't do what we do normally without volunteers. And we just have such a good reputation in the community that these are volunteers that have just been with [us] forever.*

**COVID-19 social distancing data collection protocol also serves to mitigate non-pandemic challenges to in-person survey collection.** In response to moderator guide questions about strengths of the current participant-level data collection protocol, and what interviewees anticipate would change once “COVID-19 is over,” many focused on “staying the course” with the current system. Though the telephonic proctored survey protocol was implemented in response to COVID-19 precautions and limitations on face-to-face time with customers, there are a myriad of additional benefits to telephonic proctored surveys. Volunteers shared that they often had to explain survey questions to customers, that they helped think through how to remember answers to questions about FV consumption (e.g., food frequency questions), and that “some of the survey could be tricky if you didn’t read well, or even couldn’t see the screen or page very well.” HTFMM staff and customers suggested that because of the weather in Wisconsin, many people would not want to “stand in the cold any longer than they have to, just to take a survey” and that given many of the customers were older adults, there are issues with mobility and standing for long periods of time. One customer shared:

ORIGINAL UNEDITED MANUSCRIPT

*After I get my groceries at the market, walking across the parking lot to get there, walking through the market, paying and all that – I'm spent and that's it for the day for me.*

In these cases, a subsequent telephone call for survey data collection proved beneficial to substantially decrease participant burden. When queried as to what it might look like to eliminate the HTFMM staff role and just offer a sign-up sheet for those willing to complete a subsequent survey, HTFMM evaluation experts, staff, and volunteers all agreed that would greatly limit the number of surveys completed. Staff shared their creative "sales-pitch" strategy for asking customers to agree to a post-shopping telephone call. They suggested "just being friendly" and "helping them [customers] with their grocery bags" can be very effective recruitment strategies to get participants to fill out surveys. The volunteers especially emphasized that their role is far easier because of the "warm handoff" method, and since they usually call customers within a few days of their shopping experience, most remember having agreed to completing the survey; remember the friendly, helpful, and personal contact with the staff member; and are more willing to complete the telephonic survey. One volunteer explained:

*Well, each week, we have a spreadsheet that's maintained on the server that we can access. [NAME] puts all of the potential candidates that they've gleaned from the last few days of the market operating. So they're getting the phone numbers for us already. So it's nice that we're not cold-calling people. It's a little easier when you're calling someone to do a survey and they already know it upfront. I don't have to try and convince the person, "Hey, are you willing to do this survey?"*

ORIGINAL UNEDITED MANUSCRIPT

Finally, HTFMM staff, volunteers, and customers suggested that the personal contact, whether it was helping carry groceries from the mobile market to one's car or apartment or facilitating subsequent telephone conversations to "break up the day," were especially important during COVID-19 where many of the customers conveyed that they felt isolated and lonely. This personal contact adhered to social distancing guidelines for COVID-19 precautions as all interactions were outside or via telephone. Consistent across all types of interviewees, ideas about providing a weblink or QR code to complete the survey online independently, using paper surveys, or offering onsite (e.g., at the HTFMM) surveys did not seem as efficient or effective as their current protocol. Additionally, most HTFMM customers, staff, volunteers, and leadership did not think offering a stipend (e.g., gift card) for survey completion would be necessary and thought it would be logistically complex to disseminate. Together, these findings elucidate the facilitators and strengths of HTFMM's innovative and effective GusNIP participant-level data collection protocol and implementation.

## DISCUSSION AND CONCLUSION

The four key themes as constructed across all interviews highlight the strengths and facilitators of HTFMM's successful participant-level data collection efforts throughout the COVID-19 pandemic. Literature supports the use of a strengths-based approach to public policy and program evaluation (23,24), and the theory of positive deviance suggests the importance of examining situations where an uncommon, but desirable, strategy or solution is successfully

ORIGINAL UNEDITED MANUSCRIPT

employed despite facing similar challenges as peers (25,26). In light of this theoretical and literature-based support, findings from this study can inform best practices and guide other community-based organizations who face barriers to collecting participant-level survey data. Key strengths and facilitators are central to all four themes: collaboration across multiple unique roles among HTFMM team, prioritization of resources to support evaluation, leveraging longstanding relationships, and recognizing the importance of alleviating participant burden through accommodating data collection methods. Researchers believe the nuance between two themes made it important to delineate findings into four themes – notably the first and third theme. Not only was collaboration across multiple team members with unique roles crucial to successful participant-level survey data collection (theme #1), but the unique, longstanding relationships Hunger Task Force has built over many years of community service (theme #3) led to robust volunteer and customer engagement. Together, these two themes exemplify the synergy of multiple HTFMM team member roles within the context of a long-standing, trusting, service-based environment – which led to successful participant-level data collection.

A robust body of literature examines how telephonic survey data collection compares to internet-based or paper-and-pencil survey data collection (27,28). Cost-effectiveness studies suggest telephonic survey collection to be the most expensive and highest "labor" investment (29,30), but responsiveness and recruitment efficacy literature suggests telephonic surveys yield higher response rates (30). The HTFMM audience is largely older adults who experience limited income. Therefore, internet-based survey data collection likely wouldn't align with this audience, given challenges with accessing the internet, digital literacy, and concerns with vision (e.g., to read a survey) (27). Additionally, because HTFMM utilizes a volunteer-based "workforce" for

ORIGINAL UNEDITED MANUSCRIPT

telephonic survey data collection, the relative cost of this method is minimal. Finally, HTFMM uses a "warm handoff" method for telephonic survey data collection, wherein HTFMM staff members recruit customers in person and volunteers aim to call each recruited customer within several days of this in-person recruitment. It is well established that "cold calling" for survey data collection does not yield favorable response rates, and HTFMM's innovative use of a timely staff-to-volunteers handoff method mitigates this barrier (28,31).

There are several noteworthy limitations in this study design. As a result of customer recruitment strategy, researchers did not speak with HTFMM customers who elected not to participate in the participant-level survey, as volunteers who proctored these telephonic surveys served as the agents for recruitment (e.g., at the end of a survey call, customers were invited to participate in a subsequent interview with researchers). Therefore, there may be a potential social desirability bias for the customers who participated in this study. Additionally of note, HTFMM customers are largely older adults; therefore, this successful participant-level data collection experience may be most relevant to older adults as a priority audience.  It may be with a younger audience that self-administered online survey data collection would be equally as successful. Though the sample size is small, researchers are confident saturation was reached for customer-level interviews (16), and all HTFMM leadership (100%) and almost all relevant staff and volunteers (75% and 85%, respectively) were included in the sample. The strength of single, instrumental case study methodology privileges the synergy of multiple vantage points to any given topic, in this case, HTFMM participant-level survey data collection. In conclusion, these findings demonstrate the importance of qualitative research to inform best practices, and this case study exemplifies innovative efforts to diversify and amplify recruitment efforts for survey data

ORIGINAL UNEDITED MANUSCRIPT

collection. This case study outlines an excellent example of how a community-based organization was able to overcome challenges related to data collection during a pandemic and contributes to a growing body of literature as to best practices and approaches to collecting participant-level data to evaluate public health nutrition programs.

**Acknowledgements:** We acknowledge the generous time and insight of our interviewees.

**Statement of Authors' Contributions**

1. designed research (project conception, development of overall research plan, and study oversight). **SAS, HF, AY, CP**
2. conducted research (hands-on conduct of the experiments and data collection). **SAS, CP**
3. provided essential reagents, or provided essential materials (applies to authors who contributed by providing animals, constructs, databases, etc., necessary for the research). **CP**
4. analyzed data or performed statistical analysis. **SAS, HF**
5. wrote paper (only authors who made a major contribution). **SAS, CP, AY, CBS, HF**
6. had primary responsibility for final content. **SAS**
7. other (use only if categories above are not applicable; describe briefly).
8. All authors have read and approved the final manuscript. **SAS, CP, AY, CBS, HF**

**Implications for Policy and Practice**

- First, collaboration across multiple roles on the implementation team (e.g., staff, volunteers, leadership) is essential to distribute the workload and effort needed to collect participant-level evaluation data.

ORIGINAL UNEDITED MANUSCRIPT

- Second, resources for evaluation should be prioritized in community-based, grant-funded programs and be allowable costs with the grant guidance.
- Third, partnerships between community-based organizations, academic institutions, and community-based volunteers can be effective evaluation collaborations.
- Fourth, telephonic, proctored survey data collection may be effective and may mitigate participant burden and accommodate COVID-19 social distancing safety guidelines.

ORIGINAL UNEDITED MANUSCRIPT

**Table 1**. Moderator guides used for qualitative individual interview data collection

| Participant Type | Primary Interview Questions | Probes | Rationale for Question |
|---|---|---|---|
| **HTFMM leadership, evaluation experts, staff, and volunteers** | Tell me about the Hunger Task Force Mobile Market (HTFMM). | Audience served, funding, duration, stocking, marketing | Overall context for the HTFMM |
| | Tell me about your role with the HTFMM. | Expertise, title, daily tasks, duration | Context for positionality in HTFMM |
| | Tell me about COVID-19 effects on HTFMM. | Changes, challenges, lessons learned, strengths | Understand standard operating procedure vs. COVID-19 operating procedures |
| | Walk me through your role in the surveys customers take after using HTFMM. | Recruitment, time needed, training | Understand positionality of each key participant in HTFMM participant-level evaluation |
| | After COVID-19, what do you think the HTFMM customer survey data collection process will look like? | Recruitment, survey completion, customer interest | Understand perspectives as to what 'post COVID-19' data collection could include |
| **HTFMM leadership and evaluation experts only (additional interview questions)** | If another GusNIP grantee asked you for advice on how to continue operations and evaluation of their program during COVID-19, what would you say? | Resources needed, strengths, challenges and solutions | Understand how key HTFMM informants explain their program evaluation success during COVID-19 |
| | What resources would have been / would be helpful to support your COVID-19 adapted program evaluation? | Resources available, resources lacking, resources needed | Understand how best to support GusNIP grantees with participant-level data collection |

ORIGINAL UNEDITED MANUSCRIPT

| | | | |
|---|---|---|---|
| | Tell me about an aspect of your Hunger Task Force Mobile Market COVID-19 program evaluation of which you are especially proud. | N/A | Strengths- and assets-based approach |
| **HTFMM customers** | Tell me what healthy eating means to you. | Content, frequency, health concerns | Opening question to build confidence for interviewee |
| | There are many new resources being developed across the US to help folks gain greater access to fresh, healthy food. The Hunger Task Force Mobile Market is a very unique program. Can you tell me about your experience using this Mobile Market? | Prices, food quality, convenience, budget, health | Contextualize customer experience with HTFMM |
| | I understand that just like today's telephone call with me, someone from the Hunger Task Force Mobile Market called you to complete a survey. Can you tell me about that? | Challenges, time it took, what motivated them to participate | Understand customer perspective of survey-based program evaluation |
| | Sometimes it's hard to get people to take the time to complete surveys, or even to participate in a phone call like we're doing now. Can you share any ideas of how we can get more customers of the Mobile Market to complete these surveys? | Incentives, gift cards, stipends, length, reward | Understand customer recommendations to improve engagement in program evaluation |
| | The people who developed the Hunger Task Force Mobile Market are interested in learning from customers about completing surveys for evaluating the Mobile Market. What are your thoughts on things like | Frequency, length, format taking survey, incentives | Continuing improvement for program evaluation procedures |

ORIGINAL UNEDITED MANUSCRIPT

| | | | |
|---|---|---|---|
| | how often you would be willing to take a survey or how long it should be as not to be inconvenient for a customer? | | |

ORIGINAL UNEDITED MANUSCRIPT

**Table 2.** Key roles, responsibilities, and resources needed in HTFMM Nutrition Incentive program evaluation

| Key Role | Responsibilities | Responsibility Frequency | Time and Training | Notes |
|---|---|---|---|---|
| **HTFMM Evaluation Expert** | • Set up data collection protocol<br>• Manage volunteer and staff schedules<br>• Train all volunteers and staff<br>• Facilitate communication across evaluation team<br>• Manage recruitment / data collection spreadsheet<br>• Data cleaning, data analysis<br>• Reporting | Throughout | • Set up period ~1 month at 20 hours/week<br>• Training period ~2 months at 10 hours/week<br>• Ongoing communication, scheduling, data cleaning ~weekly at 2 hours/week<br>• Manage recruitment / data collection spreadsheet ~weekly at 1 hour/week<br>• Analysis and reporting ~ as needed (e.g., end of year reporting) at 20 hours/week | • Has training in International Development, background in public policy analysis and is full-time employee at HTFMM<br>• Is bilingual (Spanish / English) and reviews all bilingual materials<br>• Proctors customer telephone surveys as there are no bilingual volunteers |
| **Academic Partner – Evaluation Expert** | • Establish additional funding evaluation purposes (e.g., CITI, software, advertising)<br>• Advise protocol for data collection<br>• Secure Institutional Review Board approval<br>• Advise for data analysis and reporting | For protocol set up and analysis | • PhD-level evaluation and marketing expert<br>• 4+ meetings with HTFMM leadership / evaluation team | • Has collaborated with HTFMM since 2017 on additional projects and has long-standing history of contribution to evaluation efforts |
| **HTFMM Leadership** | • Strategic planning<br>• Management of HTFMM activities<br>• Hire full-time evaluation expert<br>• Continue collaboration with academic expert | Throughout | • Ongoing | |

ORIGINAL UNEDITED MANUSCRIPT

| | | | | |
|---|---|---|---|---|
| | • Weekly "hands on" experience in the field with customers of HTFMM | | | |
| **HTFMM Staff** | • Assist customers at HTFMM with shopping (e.g., logistics of entering/exiting the mobile market)<br>• Educate customers on food assistance programs available<br>• Enroll customers in food assistance programs as needed<br>• Recruit customers to participate in telephonic, proctored GusNIP survey (e.g., collect telephone numbers and schedule)<br>• Enter interested customer information into recruitment / data collection spreadsheet | Daily | • Standard HTF staff training<br>• CITI training (for customer recruitment)<br>• Scripted recruitment training by HTFMM evaluation expert<br>• Weekly at HTFMM – per stop ~ 90 minutes * 1-4 stops / week + ~30 minutes spreadsheet update per stop per staff member (total across all staff members 6-10 stops / week) | • At the time of data collection HTFMM had 3 staff members who shared this responsibility<br>• Two staff members were bilingual (Spanish / English) |
| **HTFMM Volunteers** | • Refer to recruitment / data collection spreadsheet to determine which customers need to be called during any given volunteer "shift"<br>• Track telephone call reach-outs<br>• Make telephone calls to customers to proctor participant-level GusNIP surveys<br>• Enter proctored data | Weekly | • Standard HTF volunteer training<br>• Onboarding with HTFMM evaluation expert at 2 hours prior to first volunteer shift<br>• Stay abreast of e-mail updates, 'cheat sheet' documents (e.g., locations of HTFMM)<br>• Shifts weekly at 3 hours / week<br>• Each proctored | • At the time of this data collection there were 4 HTFMM volunteers participating in GusNIP survey telephone calls<br>• Each volunteer had been with HTF for many years (average 12 years) with other non-survey-proctoring responsibilities<br>• Volunteers were |

ORIGINAL UNEDITED MANUSCRIPT

| | | | | |
|---|---|---|---|---|
| | directly into Qualtrics<br>• Communicate with HTFMM evaluation expert if participant has questions, concerns, needs related to HTFMM or food aid that are ‘off script’ from the GusNIP survey | | survey ~15 minutes; most complete ~5-8 surveys per shift | provided a HTFMM cell phone but use their own computer and Internet access |

ORIGINAL UNEDITED MANUSCRIPT

**Figure 1**. Images of Hunger Task Force Mobile Market. Photo Credit: Hunger Task Force, Marketing Department, Milwaukee, WI, USA.

ORIGINAL
MANUSCRIPT

## Notes

The Nutrition Incentive Program Training, Technical Assistance, Evaluation, and Information Center (NTAE) is supported by Gus Schumacher Nutrition Incentive Program grant no. 2019-70030-30415/project accession no. 1020863 from the USDA National Institute of Food and Agriculture.

Author Disclosure: Stotz, Fricke, Perra, Byker-Shanks, Yaroch: No conflicts of interest.

Financial Disclosure: None of the authors have any financial relationship related to this submitted work.

Human Participant Compliance Statement: Ethical approval of this research was obtained from the University of Nebraska Medical Center on March 4, 2021 (IRB # 829-20-EX).

Abbreviations used: COVID-19, novel coronavirus disease of 2019; FV, Fruit and vegetables; HTFMM, Hunger Task Force Mobile Market; GusNIP, The Gus Schumacher Nutrition Incentive Program; NTAE, National Training, Technical Assistance, Evaluation, and Information Center; NI, Nutrition Incentive; USDA, United States Department of Agriculture.

